# Advances in clinical research on pharmacological management of chemotherapy-induced constipation in gastrointestinal tumor: A perspective

**DOI:** 10.1097/MD.0000000000040137

**Published:** 2024-10-18

**Authors:** Jin-Qiang Zhang, Peng-Fei Zhang

**Affiliations:** a First Ward of General Surgery Department, The First Hospital of Yulin, Yulin, China.

**Keywords:** advance, chemotherapy, constipation, gastrointestinal tumor, herbal medicine

## Abstract

Gastrointestinal tumors, including those of the stomach, colon, rectum, and esophagus, present significant global health challenges. Chemotherapy, essential for treating these cancers, often causes constipation, adversely affecting patients’ quality of life. This study examines the mechanisms behind chemotherapy-induced constipation, such as the direct impact of chemotherapeutic drugs on intestinal function, reduced fluid intake, decreased physical activity, opioid use, and psychological stress. While traditional treatments like stimulant and osmotic laxatives are commonly used, emerging therapies such as 5-HT4 receptor agonists and probiotics show promise. Traditional Chinese medicine offers additional strategies with herbal remedies and dietary adjustments. Future research should prioritize precision medicine, combining pharmacological and non-pharmacological approaches, and developing innovative therapeutics utilizing biologics and nanotechnology. Ongoing research is crucial for improving chemotherapy-induced constipation management, aiming to enhance treatment outcomes and the quality of life for chemotherapy patients with gastrointestinal tumors.

## 1. Introduction

Gastrointestinal (GI) tumors, encompassing malignancies of the stomach, colon, rectum, and esophagus, represent a significant burden on global health.^[1,2]^ Recent epidemiological data indicate that GI tumors account for a substantial proportion of cancer incidence and mortality worldwide.^[1,2]^ Colorectal cancer, for instance, is one of the most commonly diagnosed cancers and a leading cause of cancer-related deaths.^[3]^ The rising prevalence of these tumors underscores the urgent need for effective therapeutic strategies.

Chemotherapy remains a cornerstone in the treatment of GI tumors, either as an adjuvant to surgical intervention or as a primary treatment modality for advanced and metastatic cases.^[4]^ Common chemotherapeutic agents used in GI cancers include fluoropyrimidines (such as 5-fluorouracil), platinum-based compounds (like oxaliplatin), and newer targeted therapies.^[4,5]^ Despite its efficacy in tumor control and patient survival, chemotherapy is often accompanied by a range of adverse effects, particularly GI toxicities.^[5]^

One of the most prevalent and distressing GI side effects of chemotherapy is constipation.^[6,7]^ Chemotherapy-induced constipation (CIC) can result from various mechanisms, including direct effects on the GI mucosa, alterations in the enteric nervous system, reduced physical activity, and the use of concomitant medications like opioids for pain management.^[6,7]^ This condition is not only uncomfortable for patients but can also lead to more severe complications such as bowel obstruction, fecal impaction, and exacerbation of cancer-related fatigue.

The impact of constipation on the quality of life (QoL) in cancer patients cannot be overstated.^[8]^ CIC significantly affects daily functioning, emotional well-being, and overall health status. Patients may experience abdominal pain, bloating, and a constant feeling of incomplete evacuation, which can interfere with their ability to maintain normal activities and adhere to their cancer treatment regimens.^[7]^ The psychological burden of managing persistent constipation adds to the overall stress and anxiety associated with cancer diagnosis and treatment.^[7,8]^

Given the profound impact of CIC on patient outcomes, there is an urgent need to explore effective pharmacological interventions that can alleviate this condition without compromising the efficacy of cancer treatments.^[9,10]^ Current management strategies for CIC include lifestyle modifications (such as increased fiber intake and physical activity), use of laxatives (osmotic, stimulant, or stool softeners), and newer agents like prokinetics and secretagogues.^[9,10]^ However, these treatments vary in effectiveness, and some may carry additional side effects or interact with ongoing chemotherapy.

The pursuit of optimal pharmacological therapies for CIC is crucial for several reasons.^[11,12]^ Firstly, effective management of constipation can enhance the overall well-being and comfort of cancer patients, allowing them to focus more on recovery and less on the distress of GI side effects. Secondly, by mitigating the adverse effects of chemotherapy, patients are more likely to adhere to their prescribed treatment regimens, potentially improving overall cancer outcomes. Thirdly, timely and effective management of constipation can prevent severe complications such as bowel obstruction, which may necessitate surgical intervention and further complicate the patient’s health status. Lastly, reducing the incidence and severity of CIC can decrease healthcare costs associated with managing chemotherapy side effects, including hospital admissions for severe constipation-related complications.

In summary, advancing our understanding of the pharmacological management of CIC in GI tumor patients is imperative. This review aims to summarize the latest clinical research progress in this area, focusing on the efficacy and safety of various therapeutic agents. Through comprehensive analysis, we hope to identify promising treatments that can be integrated into clinical practice to improve patient care and outcomes.

## 2. Clinical trials and research design

### 2.1. Study types

#### 2.1.1. Observational studies

Essential for gauging the real-world effectiveness and safety of treatments for CIC, observational studies track patients in their usual environments without interference. These studies are invaluable for observing natural treatment effects, adherence, and long-term impacts. Typical designs include cohort studies, which monitor groups over time to assess treatment outcomes, and case–control studies, which contrast patients with and without constipation to uncover risk factors and effects of treatments. For example, one study demonstrated that the topical application of Da Huang Wan at the Shenque acupoint significantly alleviated constipation symptoms in gastric cancer patients, with an effectiveness rate of 84.78%^[13]^ (Table 1).

**Table 1:** Clinical studies of pharmacological management of constipation chemotherapy-induced constipation in gastrointestinal tumor.

Study	Patients	Sample size	Publication type	Treatment	Main findings
Wen P. (2018)^[13]^	Patients with gastric cancer	46	Observation study	Dahuang Wan	Applying Da Huang Wan to the Shenque acupoint can alleviate constipation symptoms in gastric cancer patients after chemotherapy, with an efficacy rate of 84.78%
Li GB. (2023)^[14]^	Patients with colon cancer	400	Randomized controlled trial	Liu Jun An Wei Formula (LJAW)	LJAW significantly improves gastrointestinal reactions during adjuvant chemotherapy for colon cancer, with factors such as initial diarrhea status, grouping, age, gender, and cancer stage influencing its effectiveness on symptoms like diarrhea, constipation, vomiting, and nausea
Han CY. (2023)^[15]^	Patients with esophageal cancer	138	Randomized controlled trial	Jiawei Xiangsha Liujunzi Decoction	Oral administration of Jiawei Xiangsha Liu Junzi Decoction, in addition to standard radiotherapy and chemotherapy care, can effectively reduce nausea, vomiting, and constipation caused by the treatments, thereby improving the quality of life for esophageal cancer patients

#### 2.1.2. Randomized controlled trials (RCTs)

Regarded as the gold standard for clinical research, RCTs meticulously assess the efficacy and safety of treatments by randomly assigning patients to treatment or control groups, thereby minimizing bias. This method facilitates direct comparisons between interventions and control conditions, such as a placebo or standard care, and provides high-quality evidence on the effectiveness of treatments ranging from traditional laxatives to innovative drugs and complementary therapies like traditional Chinese medicine (TCM). Significant findings from RCTs include improvements in GI reactions during adjuvant chemotherapy for colon cancer with the Liu Jun An Wei Formula^[14]^ and enhancements in QoL for esophageal cancer patients treated with Jiawei Xiangsha Liujunzi Decoction during chemoradiotherapy^[15]^ (Table 1).

### 2.2. Outcome measures

#### 2.2.1. Improvement in constipation symptoms

The primary endpoint in trials for constipation is the enhancement of bowel movement frequency and stool consistency, measured objectively with tools such as the Bristol Stool Form Scale and patient diaries that log bowel habits.^[16–19]^

#### 2.2.2. QoL assessments

Given the substantial impact of constipation on patients undergoing chemotherapy, assessing changes in QoL is critical. This is typically done using validated questionnaires like the Patient Assessment of Constipation-QoL,^[20,21]^ which explores various dimensions of well-being including physical, psychological, and social aspects.

#### 2.2.3. Recording adverse reactions

Monitoring and documenting adverse reactions is crucial for assessing the safety profile of constipation treatments.^[20]^ This includes tracking everything from mild GI discomfort to serious systemic effects, ensuring treatments are both effective and safe.

### 2.3. Research challenges

#### 2.2.1. Patient adherence

A significant challenge in clinical trials is ensuring that patients adhere to treatment protocols, especially as those undergoing chemotherapy may experience multiple adverse effects which could deter their participation in a strict study regimen.^[22]^ Enhancing adherence involves simplifying treatment schedules, providing detailed patient education, and supporting patients throughout the duration of the trial.

#### 2.2.2. Long-term efficacy and safety

Evaluating the long-term efficacy and safety of constipation treatments poses significant challenges.^[23]^ Although short-term studies provide data on immediate benefits and adverse effects, understanding the sustained efficacy and potential long-term adverse reactions requires extended follow-up and continuous patient engagement, often difficult to maintain in long-term studies.^[23]^

#### 2.2.3. Variability in patient populations

The diverse nature of cancer patient populations in terms of demographics, types of cancer, and treatment approaches introduces variability that complicates the analysis and interpretation of research outcomes.^[16]^ Addressing this involves stratifying study participants by relevant demographic and clinical factors and employing large, representative sample sizes to enhance the reliability and applicability of findings.

In summary, designing clinical trials and research on CIC requires careful consideration of study types, outcome measures, and the challenges inherent in such studies. Observational studies and RCTs each provide critical insights into the efficacy and safety of treatments. Key measures include evaluating improvements in constipation symptoms, QoL, and the recording of adverse reactions. Overcoming challenges such as ensuring patient adherence, evaluating long-term effects, and managing variability among patient populations is essential for advancing our understanding and treatment of CIC, ultimately aiming to improve patient outcomes and QoL.

## 3. Mechanisms of CIC

### 3.1. Impact of chemotherapeutic drugs on intestinal function

Chemotherapeutic agents can significantly disrupt normal intestinal function, leading to constipation through various mechanisms.^[11,12]^ Firstly, these drugs often have a direct effect on the smooth muscle of the GI tract. Many chemotherapeutic agents can induce myopathy or neuropathy in the enteric nervous system, which governs gut motility.^[11]^ This disruption can result in decreased peristalsis, leading to delayed bowel movements and constipation.

Additionally, chemotherapeutic drugs can alter neurotransmitter levels and activity involved in GI motility.^[11,12]^ For example, certain chemotherapeutic agents can reduce levels of acetylcholine, a critical neurotransmitter that stimulates muscle contractions in the digestive tract. Inhibiting acetylcholine or other excitatory neurotransmitters can reduce the contractile activity of the intestines, contributing to constipation.

### 3.2. Patient factors

Several patient-related factors also contribute to the development of CIC.^[9,24–26]^ One significant factor is the reduction in fluid and dietary intake. During chemotherapy, patients often experience nausea and vomiting, leading to dehydration and decreased oral intake.^[9]^ Insufficient fluid intake can result in harder stools, making them more difficult to pass. Similarly, a diet low in fiber can contribute to constipation by reducing stool bulk and softness.

Moreover, chemotherapy often leads to reduced physical activity.^[24,26]^ Fatigue and weakness are common side effects of chemotherapy, limiting a patient’s ability to stay active. Physical inactivity is a well-known risk factor for constipation because regular movement stimulates bowel activity. When patients are less active, GI motility can slow down, leading to constipation.

### 3.3. Other related factors

Beyond the direct effects of chemotherapy and patient behavior, several other factors can exacerbate constipation in cancer patients.^[27]^ One such factor is the presence of comorbid conditions and complications. Cancer patients often suffer from multiple health issues, such as diabetes, hypothyroidism, or hypercalcemia, which impair bowel function.^[27]^ The management of these conditions often involves medications that can further contribute to constipation.

The use of concomitant medications, especially opioids for cancer-related pain, significantly contributes to constipation.^[28]^ Opioids bind to receptors in the GI tract, reducing bowel motility and increasing water absorption from the stool, leading to harder and less frequent stools. This opioid-induced constipation can be particularly severe and difficult to manage.

Another relevant factor is the psychological stress associated with cancer and its treatment.^[29]^ Anxiety, depression, and stress are common among cancer patients and can affect GI function through the gut–brain axis. Psychological distress can alter gut motility and secretion, contributing to the development of constipation.

In summary, CIC is a multifactorial condition resulting from the direct effects of chemotherapeutic drugs on the GI system, patient-related factors such as reduced fluid and dietary intake and decreased physical activity, and other related factors including comorbid conditions, concomitant medications, and psychological stress. Understanding these mechanisms is essential for developing effective strategies to prevent and manage constipation in cancer patients. Comprehensive approaches that address these various factors are needed to improve the QoL and treatment outcomes for patients undergoing chemotherapy.

## 4. Current research on pharmacological treatment of constipation

### 4.1. TCM in treating constipation

TCM offers a holistic approach for managing constipation, incorporating herbal remedies, and dietary adjustments. TCM is based on principles aimed at balancing the body’s *Qi* (vital energy), blood, and fluids.^[30]^

#### 4.1.1. Herbal remedies

Several Chinese herbal formulations are effectively used to treat constipation. For instance, Ma Zi Ren Wan, containing hemp seed, rhubarb, and other herbs, is used to lubricate the intestines and promote bowel movements. Clinical studies indicate that this and other TCM formulations can effectively alleviate constipation symptoms, especially when conventional treatments are ineffective or cause significant side effects.^[30]^

#### 4.1.2. Dietary adjustments

TCM emphasizes the importance of diet in maintaining GI health. Dietary recommendations are tailored based on the patient’s specific symptoms and constitution, often incorporating high-fiber foods like vegetables and fruits to support bowel movements, while avoiding greasy and overly processed foods.^[17]^

#### 4.1.3. Potential interactions with antitumor treatments

TCM offers supportive care for managing constipation and other chemotherapy-induced side effects.^[18,31]^ However, integrating TCM with conventional cancer treatments requires careful monitoring due to potential interactions that might affect the safety and efficacy of chemotherapeutic agents.^[18,31]^ Certain herbal components in TCM can influence the metabolism of chemotherapeutic drugs by affecting key enzymes, such as cytochrome P450.^[32]^ For instance, compounds found in St. John Wort may accelerate drug metabolism, potentially reducing therapeutic effectiveness.^[33]^ Conversely, agents in grapefruit juice can inhibit enzyme activity, increasing drug toxicity.^[34]^ Antioxidants present in some herbs could theoretically interfere with the mechanisms through which certain chemotherapies exert their effects, potentially altering the intended outcomes of the treatment.^[35]^ To manage these risks, healthcare providers must thoroughly review any TCM supplements taken by patients, making necessary adjustments to treatment plans to ensure compatibility and safety. Patients should be educated about the potential risks of combining herbal remedies with chemotherapy and encouraged to discuss all herbal and dietary supplement use with their healthcare team to avoid harmful interactions. Enhanced research into the interactions between TCM and chemotherapeutic agents will aid in developing robust guidelines for safely incorporating herbal medicine into cancer care.^[36]^ By carefully balancing TCM and conventional treatments, we can ensure that TCM supports rather than compromises the efficacy of cancer therapies.^[36]^ This dual approach not only ensures safety but also enhances the quality of life for patients undergoing rigorous cancer treatments. This strategy aligns with the holistic principles in advancing clinical research in TCM, emphasizing a comprehensive approach that integrates traditional and modern medical practices to improve patient outcomes.

### 4.2. Traditional drug therapies

The management of constipation, particularly in cancer patients undergoing chemotherapy, has historically relied on several classes of traditional medications.^[11,37,38]^ Each class operates through different mechanisms to alleviate symptoms and improve bowel movements.

#### 4.2.1. Stimulant laxatives

Agents such as senna and bisacodyl fall into this category. These drugs stimulate the enteric nerves, increasing intestinal motility to induce bowel movements.^[37,38]^ While effective, they can cause cramping and discomfort, and their long-term use is generally discouraged due to potential colonic damage and laxative dependence.

#### 4.2.2. Osmotic laxatives

Examples include polyethylene glycol, lactulose, and magnesium citrate. These drugs draw water into the intestinal lumen, softening the stool and promoting bowel movements.^[37,38]^ Osmotic laxatives are typically well-tolerated and effective; however, they can cause bloating and electrolyte imbalances in some patients.

#### 4.2.3. Stool softeners and lubricants

Docusate sodium, a commonly used stool softener, increases water absorption by the stool, making it easier to pass. Mineral oil, a lubricant, coats the stool and intestinal lining, preventing water absorption and easing stool passage. These treatments are effective for mild constipation but are limited in severe cases and are not recommended for long-term use due to potential side effects like fat-soluble vitamin malabsorption.^[37,38]^

### 4.3. Emerging drug therapies

Recent research focuses on developing new pharmacological agents that target specific mechanisms of constipation, offering potential advantages over traditional therapies.^[19,39,40]^

#### 4.3.1. 5-HT4 receptor agonists

Prucalopride is a notable example.^[19]^ These drugs enhance the release of acetylcholine and calcitonin gene-related peptide in the gut, promoting peristalsis and improving bowel function. Clinical trials have demonstrated the efficacy of prucalopride in treating chronic constipation, including in chemotherapy-induced cases, with a favorable safety profile.^[19]^

#### 4.3.2. Highly effective microbial preparations

Probiotics and prebiotics are increasingly studied for their potential to treat constipation by modulating the gut microbiota.^[39,40]^ Specific strains of probiotics, such as *Bifidobacterium* and *Lactobacillus*, have shown promise in clinical trials for improving bowel movements and stool consistency. These agents work by enhancing the gut flora balance, producing short-chain fatty acids that stimulate colonic activity, and improving overall GI health.

#### 4.3.3. Peripherally acting µ-receptor antagonists (PAMORAs)

In addressing opioid-induced constipation among cancer patients, PAMORAs such as methylnaltrexone, naloxegol, and alvimopan offer a targeted therapeutic approach.^[41]^ These agents specifically antagonize opioid receptors in the GI tract, thus preventing the opioids from inhibiting intestinal peristalsis without affecting the central analgesic effects of the opioids.^[42]^ This mechanism of action is crucial as it allows the continuation of effective pain management while directly addressing constipation, a common and debilitating side effect in oncology palliative care.^[42]^ Clinical evidence supports the efficacy of PAMORAs in restoring bowel function, with these medications typically recommended for patients whose constipation has not responded to conventional laxatives.^[43,44]^ Their use significantly enhances the QoL and compliance with opioid regimens, marking them as indispensable in comprehensive cancer care strategies.^[45]^ By integrating PAMORAs into the pharmacological management of constipation, clinicians can ensure a balanced approach for treating both the symptoms and side effects of cancer therapy, thereby improving overall patient outcomes.

In summary, the pharmacological treatment of constipation in patients undergoing chemotherapy for GI tumors includes a range of traditional and emerging therapies. Traditional treatments, such as stimulant and osmotic laxatives, remain widely used. However, newer agents like 5-HT4 receptor agonists and probiotics offer promising alternatives. Additionally, TCM provides a holistic and potentially effective approach for managing constipation. Ongoing research and clinical trials continue to refine these therapies, aiming to improve efficacy and safety profiles to enhance the QoL for cancer patients.

## 5. Future research directions

### 5.1. Application of precision medicine

In the realm of managing CIC, the integration of precision medicine offers a promising avenue for future research, focusing on individualized patient care and optimized treatment outcomes.^[46–49]^

#### 5.1.1. Genetic profiling for predicting drug response

Utilizing genetic profiling to identify specific genetic variants influencing individual responses to various laxatives and treatments is a key aspect of precision medicine.^[46]^ Variations in genes associated with drug metabolism, such as cytochrome P450 enzymes, can significantly impact drug efficacy and safety profiles. Understanding these genetic variations enables clinicians to tailor treatment plans, predicting which patients are likely to benefit from specific therapies while minimizing adverse effects.

#### 5.1.2. Personalized treatment plans

Armed with genetic insights, healthcare providers can craft personalized treatment regimens tailored to each patient’s unique genetic makeup and clinical profile.^[47–49]^ This tailored approach encompasses selecting the most suitable pharmacological agents, adjusting dosages, and combining therapies to optimize efficacy and minimize side effects.^[47–49]^ By individualizing treatment plans, precision medicine aims to achieve superior management of CIC, thereby enhancing patient outcomes and QoL.

### 5.2. Comprehensive treatment strategies

Future research efforts should focus on developing comprehensive treatment strategies that integrate pharmacological and non-pharmacological interventions, recognizing the multifaceted nature of constipation management.^[23,50,51]^

#### 5.2.1. Integration of pharmacological and non-pharmacological therapies

Combining pharmacological interventions with non-pharmacological approaches, such as dietary modifications, physical activity regimens, and behavioral therapies, holds promise for holistic constipation management.^[23,50,51]^ For instance, coupling laxative therapy with dietary fiber supplementation and increased fluid intake can enhance treatment efficacy. Tailored physical exercise programs aimed at stimulating GI motility further complement pharmacotherapy. Behavioral interventions, including biofeedback and cognitive-behavioral techniques, empower patients to adopt healthy bowel habits and mitigate stressors contributing to constipation.

#### 5.2.2. Patient education and lifestyle adjustments

Emphasizing patient education on the importance of dietary habits, hydration, and regular physical activity is paramount. Providing patients with knowledge about bowel-friendly dietary choices, the significance of adequate hydration, and the benefits of maintaining an active lifestyle empowers them to actively participate in their constipation management. Through education and lifestyle adjustments, patients can develop sustainable habits conducive to optimal bowel health, thereby fostering treatment adherence and improving long-term outcomes.

### 5.3. Development of novel therapeutics

Future research endeavors should prioritize the development of innovative pharmacological agents targeting specific mechanisms underlying constipation, leveraging advancements in biologics and nanotechnology.

#### 5.3.1. Targeted mechanism-based drugs

Research initiatives should explore novel pharmacotherapeutic agents that selectively target key pathways implicated in constipation pathophysiology.^[52]^ For instance, drugs modulating serotonin receptors or other neurotransmitter systems involved in GI motility represent promising avenues for exploration. By specifically targeting aberrant mechanisms underlying constipation, these innovative therapies aim to deliver enhanced efficacy and reduced side effects compared to conventional laxatives.

#### 5.3.2. Biologics and nanotechnology applications

Harnessing the potential of biologics, such as monoclonal antibodies, and nanotechnology in drug delivery systems presents exciting prospects for constipation treatment innovation.^[53]^ Biologics offer targeted interventions by selectively binding to specific molecules implicated in GI dysmotility or inflammation. Concurrently, nanotechnology facilitates precise drug delivery, optimizing bioavailability and enabling targeted release at desired sites of action. By capitalizing on biologics and nanotechnology, researchers endeavor to advance constipation therapeutics with heightened precision and efficacy.

In summary, future research directions in the management of CIC are anchored in precision medicine, comprehensive treatment strategies, and the development of novel therapeutics. Precision medicine strives to individualize care through genetic profiling and personalized treatment plans, optimizing therapeutic outcomes. Comprehensive treatment strategies integrate pharmacological and non-pharmacological interventions, fostering holistic constipation management. Lastly, the development of novel therapeutics, leveraging targeted mechanisms, biologics, and nanotechnology, represents the forefront of innovation in constipation treatment, aiming to deliver superior efficacy and safety profiles. These concerted research endeavors aim to elevate constipation management standards, ultimately enhancing patient well-being and QoL.

## 6. Summary

Significant progress has been made in the pharmacological treatment of CIC in patients with GI tumors. Traditional treatments, such as stimulant and osmotic laxatives, remain effective but have limitations. Emerging therapies, including 5-HT4 receptor agonists and probiotics, offer promising alternatives by targeting specific mechanisms and improving gut health. TCM, with its herbal remedies and acupuncture, provides valuable complementary options.

Future research is crucial for advancing constipation management. The application of precision medicine through genetic profiling and personalized treatment plans can optimize therapeutic outcomes. Integrating pharmacological and non-pharmacological approaches, along with patient education and lifestyle adjustments, offers a holistic solution. The development of novel therapeutics, incorporating biologics and nanotechnology, represents the forefront of innovation, aiming to enhance efficacy and safety.

## Author contributions

**Conceptualization:** Jin-Qiang Zhang, Peng-Fei Zhang.

**Data curation:** Jin-Qiang Zhang, Peng-Fei Zhang.

**Investigation:** Peng-Fei Zhang.

**Methodology:** Peng-Fei Zhang.

**Project administration:** Peng-Fei Zhang.

**Resources:** Jin-Qiang Zhang, Peng-Fei Zhang.

**Supervision:** Peng-Fei Zhang.

**Validation:** Jin-Qiang Zhang, Peng-Fei Zhang.

**Visualization:** Jin-Qiang Zhang, Peng-Fei Zhang.

**Writing – original draft:** Jin-Qiang Zhang, Peng-Fei Zhang.

**Writing – review & editing:** Jin-Qiang Zhang, Peng-Fei Zhang.
